# Did Hans Asperger actively assist the Nazi euthanasia program?

**DOI:** 10.1186/s13229-018-0209-5

**Published:** 2018-04-19

**Authors:** Simon Baron-Cohen, Ami Klin, Steve Silberman, Joseph D. Buxbaum

**Affiliations:** 1 Autism Research Centre, Department of Psychiatry, Cambridge University, Douglas House, 18B Trumpington Road, Cambridge, CB2 8AH UK; 2 Marcus Austin Center, Emory University School of Medicine and Children’s Healthcare of Atlanta, Atlanta, GA 30329 USA; 3 Author, Neurotribes, New York, USA; 4 Seaver Autism Center for Research and Treatment, Department of Psychiatry, Icahn School of Medicine at Mount Sinai, New York, 10029 NY USA

In this issue of *Molecular Autism*, we publish an article by Herwig Czech, a historian of medicine at the Medical University of Vienna. His carefully researched article concludes that the pediatrician Hans Asperger, after whom the subgroup of Asperger syndrome was named, and who worked in the University of Vienna Pediatric Clinic during the Second World War, not only collaborated with the Nazis but actively contributed to the Nazi eugenics program by referring profoundly disabled children to the *Am Spiegelgrund* clinic located elsewhere in Vienna. This was a clinic that he knew participated in the Third Reich’s child euthanasia program, where children were killed as part of the Nazi goal of eugenically engineering a genetically “pure” society through “racial hygiene” and the elimination of lives deemed a “burden” and “not worthy of life.”

We take the unusual step of publishing this Editorial so as to explain our reasons for publishing this article. Two of us are Editors-in-Chief of Molecular Autism (SBC and JDB), one of us served as Action Editor during the long review process of this article (SBC), and two of us served as anonymous reviewers for this article, but have decided to forgo their anonymity (SS and AK).

We write this Editorial for two reasons. First, to assert the importance of this kind of scholarship and its relevance to this Journal, which aims to publish excellent research into autism of any kind, whether the research focuses on the molecular, neurological, psychological, clinical, or in this case social aspects. Second, to underline our support of this article for exploring in meticulous detail how a medical doctor, Hans Asperger, who for a long time was seen as only having made valuable contributions to the field of pediatrics and child psychiatry, was, as Herwig Czech’s newly unearthed evidence shows, also guilty of actively assisting the Nazis in their abhorrent eugenics and euthanasia policies. We are persuaded by Herwig Czech’s important article that Asperger was not just doing his best to survive in intolerable conditions but was also complicit with his Nazi superiors in targeting society’s most vulnerable people.

We will not repeat the evidence and main findings of Herwig Czech’s article here but will note that the conclusion concur with a new book on this topic, published in 2018, by Edith Sheffer, and entitled *Asperger’s Children: The origins of autism in Nazi Vienna*. Like Czech, Sheffer compellingly makes the case that Asperger willingly became a cog in the Nazi killing machine, referring children both directly and indirectly to *Am Spiegelgrund*. This was the clinic where children who were deemed genetically incapable of social conformity, or who had physical or psychological ‘defects’ that were deemed undesirable and assumed to be genetically determined, were killed through starvation and lethal injections, and whose deaths were recorded as due to pneumonia.

Sheffer argues that Asperger may have been sympathetic to the Nazi goal of eliminating any children who could not fit in with the *Volk*, that is, fascist collectivism and the creation of a homogeneous Aryan people. In 1938, the Dean of the Medical School removed more than half of its faculty, predominantly Jewish doctors. In contrast, at the age of 28 Hans Asperger had enjoyed premature promotion to be Director of the Curative Education Clinic.

Sheffer describes how, under Hitler’s regime, the profession of psychiatry that previously had been based on compassion and empathy became part of the eyes and ears of the Third Reich. Under the direction of Public Health Officials, professionals went into homes to index and classify every child as genetically fit or unfit, and assigning diagnostic labels that would ultimately determine who would live and who would be killed.

We want to stress our immense respect for our late colleague Dr. Lorna Wing, in London, who first coined the term Asperger syndrome in 1981 and developed her concept of the “autism spectrum” encouraged by the insights of Asperger and his staff. At that time, she and we as scientists and clinicians, as well as the broader autism community, were unaware of Hans Asperger’s close alliance with, and support of, the Nazi program of compulsory sterilization and euthanasia.

Sheffer, following Steve Silberman and John Elder Robison, also mentions the fact that Georg Frankl, a staff physician at the clinic, and the psychologist Anni Weiss, had already published on cases similar to those later described as “autistic psychopaths” before Asperger. Because Frankl and Weiss were Jewish, they were forced to leave Austria and went to the US, where they married shortly after their arrival. As Asperger's understanding of autism surely drew on their work and observations, and later helped inspire Lorna Wing to define the scope of the autism spectrum, Frankl and Weiss deserve credit for contributing to the modern understanding of autism.

The degree of Asperger’s involvement in the targeting of Vienna’s most vulnerable children has remained an open and vexing question in autism research for a long time. Some readers will remind us that many of Asperger’s mentors and colleagues were more public and vociferous of their support of Nazi racial ideology than he was. Some may identify mitigating circumstances in the possibility that he sacrificed some children to save others. Some may situate the subject matter of this article as just another example of the bioethical challenges posed by eponymous medical syndromes discovered by physicians, or scientists taking advantage of helpless children subjected to the Nazi treatment of those deemed unfit to live. We believe that the value of Czech’s scholarship is that it establishes the necessary evidentiary framework for future discussion.

